# Minimal Component, Protein‐Free, and Cost‐effective Human Pluripotent Stem Cell Cardiomyocyte Differentiation

**DOI:** 10.1002/cpz1.70099

**Published:** 2025-02-11

**Authors:** Jessika B. Iwanski, Odunayo S. Lawal, William T. Kwon, Isabella Vazquez, Jared M. Churko

**Affiliations:** ^1^ Department of Cellular and Molecular Medicine The University of Arizona Tucson Arizona; ^2^ Department of Internal Medicine UT Southwestern Medical Center Dallas Texas; ^3^ Department of Clinical Translational Sciences The University of Arizona Tucson Arizona; ^4^ BIO5 Institute The University of Arizona Tucson Arizona; ^5^ These authors contributed equally to this work.

**Keywords:** animal‐free, cardiomyocytes, hPSC‐CM, protein‐free, stem cells

## Abstract

Human pluripotent stem cell‐derived cardiomyocytes (hPSC‐CMs) have become a powerful source for the *in vitro* modeling of cardiac diseases and various other essential applications, including cardiotoxicity screening and regenerative cell replacement therapies. Although many differentiation protocols have been developed to generate cardiomyocytes from human pluripotent stem cells, these protocols are costly and complex, requiring expensive and often unnecessary components (e.g., B27 medium supplement). In addition, the use of animal‐derived growth factors limits their use for regenerative medicine purposes. To address these issues, herein, we have developed an efficient, cost‐effective, and protein‐free hPSC‐CM protocol using only two components: DMEM/F12 basal medium and l‐ascorbic acid 2‐phosphate. By eliminating xenobiotic and complex components, the efficiency of directed differentiations is increased, the variability between cardiac differentiations is decreased, and the scalability of cell production is enhanced. Adaptation of this efficient, low‐cost, and user‐friendly cardiac differentiation protocol will enrich the utility and applicability of hPSC‐CMs in drug discovery, cell therapies, tissue engineering, disease modeling, precision medicine, and cardiac regenerative medicine. © 2025 The Author(s). Current Protocols published by Wiley Periodicals LLC.

**Basic Protocol 1**: hPSC cell culture

**Basic Protocol 2**: hPSC‐CM differentiation

**Basic Protocol 3**: Characterization of hPSC‐CMs by immunofluorescence (IF) imaging

## INTRODUCTION

The adult human myocardium has limited regenerative potential following an injury or ischemic event (Hesse et al., 2018). Furthermore, obtaining primary human cardiomyocytes to study cardiovascular diseases is often very challenging due to bioavailability, the difficulty of obtaining cells, and the non‐proliferative nature of native human cardiomyocytes (Bergmann et al., 2015). The use of *in vitro* differentiation of cardiomyocytes from both human pluripotent stem cells (hPSCs) and human embryonic stem cells (hESCs), collectively termed human pluripotent stem cells (hPSCs), has provided an alternative avenue to advance our abilities to study cardiovascular biology, perform drug discovery and safety studies, and apply these cells towards regenerative medicine efforts (Kussauer et al., 2019). Over the past decade, significant progress has been made in improving hPSC‐CM differentiation protocols (Balafkan et al., 2020; Burridge et al., 2014; Guo et al., 2018; Lian et al., 2012; Tan et al., 2018). However, current methods require the addition of growth factors, exogenous proteins, and numerous complex medium components, which add variability to the gene expression signatures and consequently alter the intrinsic and functional properties of generated hPSC‐CMs. These changes may impact the downstream potential use and data interpretation of hPSC‐CMs. Furthermore, challenges arise when using a scalable system for directed hPSC‐CM differentiation, as current protocols (1) are time‐intensive and difficult to perform, (2) do not generate hPSC‐CMs with sufficient purity, (3) use costly and unnecessary reagents, and (4) use xenobiotic components. Thus, to overcome this, we have optimized the hPSC cardiomyocyte differentiation protocol to utilize minimal components, to be protein‐ and xenobiotic‐free, and to be cost‐effective. This, in turn, maximizes differentiation scalability and generates hPSC‐CMs more applicable for downstream research applications (such as transcriptomic/proteomic/metabolomic studies, functional analyses, and cellular/biochemical experimentation) without compromising the integrity and function of the hPSC‐CMs generated.

The protocols below provide step‐by‐step instructions for the preparation, generation, and evaluation of directed hPSC cardiac differentiation. Basic Protocol 1 details the process for culturing, passaging, and maintaining hPSCs prior to cryopreservation and long‐term storage of cells. The user may opt to use plates coated either with Matrigel, a mixture of extracellular matrix components that contains mouse‐derived protein components, or with vitronectin, which is xenogeneic‐free. The latter may be more desirable as it should not interfere with the gene expression profiles of hPSCs. Next, Basic Protocol 2 details the process of differentiating hPSCs into cardiomyocytes using a two‐component protocol involving l‐ascorbic acid 2‐phosphate (AA2P) and DMEM/F12 base medium alongside the implementation of the Wnt modulation system. Also discussed is the purification of hPSC‐CMs to allow the user to obtain a target goal of >80% purity, should the initial differentiation process become populated by undesirable cell populations. Lastly, Basic Protocol 3 describes the characterization of differentiated cardiomyocytes via immunofluorescence microscopy by probing for expression of cardiac‐specific proteins (e.g., cardiac α‐actinin and F‐actin).

*CAUTION*: All institutional guidelines for handling human cell lines should be adhered to and performed under the acceptance of an Institutional Review Board (IRB)‐approved protocol.

*NOTE*: All protocols should be performed in a BSL‐2 safety cabinet using proper sterile technique. Routine mycoplasma testing should be performed on each hPSC line.

*NOTE*: All protocols involving animals must be reviewed and approved by the appropriate Animal Care and Use Committee and must follow regulations for the care and use of laboratory animals. Appropriate informed consent is necessary for obtaining and use of human study material.

## Basic Protocol 1 hPSC CELL CULTURE

Before hPSC‐CM differentiation can begin, hPSCs must first be cultured, passaged, and maintained in a proliferative state. Because hPSCs do not attach or grow on plastic tissue culture‐treated plates, specific matrices, such as Matrigel and Cultrex, are commonly used to facilitate hPSC adherence and growth. However, it is important to note that Matrigel contains mouse‐derived extracellular matrix proteins, which may alter gene expression profiles of hPSCs. Therefore, to maximize xenogeneic‐free components, recombinant human vitronectin or synthetic matrices may be used as an alternative. This section describes (1) the preparation of cell culture plates (both with the commonly used Matrigel and the recommended xenogeneic‐free vitronectin), (2) the maintenance of hPSCs, and (3) the cryopreservation of cells in preparation for hPSC‐CM differentiation (Basic Protocol 2). At the end of this section, the user should be able to successfully generate cryopreserved vials of ∼1‐2 million hPSCs, ready for downstream use.

### Materials

Corning Matrigel Growth Factor Reduced (GFR) Basement Membrane Matrix (Corning, cat. no. 356231) or recombinant human vitronectin (VTN‐N; Thermo Fisher Scientific, cat. no. A14700)
Dulbecco's Modified of Eagle's Medium (DMEM/F12 50:50; Corning, 10‐092‐CM)
1× phosphate‐buffered saline (PBS; e.g., Thermo Fisher Scientific, Gibco, cat. no. 10010002)
Human embryonic stem cells (hESCs): e.g., H7 (WA07) hESCs (Wicell, Wisconsin, MA; NIHhESC‐10‐0061); or human induced pluripotent stem cells: e.g., JCAZ001, JCAZ002, JCAZ1065, or JCAZ1066 (University of Arizona iPSC Core)
70% (v/v) ethanol (diluted in water from 100% ethanol, Fisher Scientific, cat. no. 04‐355‐224)
E8 medium (Thermo Fisher Scientific; cat. no. A1517001)
Y‐27632 Rho kinase inhibitor (Tocris, cat. no. 1254)
Cell Dissociation Buffer (CDB; Sigma Aldrich, 13151014)
Bambanker medium (Lymphotec Inc., cat. no. 302‐14681)

Ice bucket
1000‐, 200‐, 20‐, and 10‐µl pipets (Fisher Scientific, cat. no. 14‐285‐904)
Corresponding pipet tips (USA Scientific, cat. nos. 1111‐2020, 1110‐1000, 1111‐3000, and 1120‐1710)
–80°C freezer (Thermo Fisher Scientific, cat. no. TSX60086D)
6‐well flat‐bottom culture plates (Greiner Bio‐One CELLSTAR, cat. no. 07‐000‐208)
1300 Series Class II, Type B2 Biological Safety Cabinets (Thermo Fisher Scientific, cat. no. 1310TS1)
15‐ml conical tubes (USA Scientific, cat. no. 5618‐8271)
DualSpin Rotor Centrifuge (Thermo Fisher Scientific, cat. no. 75008810)
Heracell VIOS 160i CO_2_ Incubator (Thermo Fisher Scientific, cat. no. 50144906)
Inverted phase‐contrast microscope (Nikon ECLIPSE Ts2)
Hemocytometer (Fisher Scientific, cat. no. 02‐671‐6)
Cryovials (Corning, cat. no. 430488)
Liquid nitrogen tank (Jencon K Series)

### Preparation of Matrigel‐coated tissue culture plates

1a Thaw growth‐factor‐reduced Matrigel (∼10 mg/ml) in a 4°C refrigerator overnight. Prepare 350‐µl aliquots of Matrigel in sterile 1.5‐ml sterile microcentrifuge tubes on ice. Store aliquots in a –80°C freezer until ready for use.
  Matrigel must be kept on ice at all times to prevent solidification and should not undergo multiple freeze‐thaw cycles.
  For a 10‐ml bottle of Matrigel, 28 aliquots will be generated.
2a The day before coating culture plates, remove a Matrigel aliquot from the –80°C freezer and thaw at 4°C.
3a Once thawed, add 1 aliquot of Matrigel to 50 ml ice‐cold DMEM/F12 or 1× PBS.
  Given that PBS is not as expensive as other buffers, it is preferable to dissolve Matrigel in PBS rather than DMEM/F12.
  Do not allow the Matrigel solution to warm to 12°C before coating. In addition, the Matrigel must remain on ice throughout the coating process.
4a Add 2 ml/well of Matrigel mixture to a 6‐well plate.
  Adjust volumes accordingly for different plate/well surface areas. Slowly rock the culture plate to completely cover the bottom of the well with Matrigel mixture.
5a Allow plates to rest for at least 16 hr at 4°C or 1 hr at room temperature (21°C) before use.
6a Coated plates may be sealed with Parafilm to prevent dehydration and stored up to 10 days at 4°C or up to 4 days at 37°C.

### Preparation of vitronectin‐coated tissue culture plates

1b Alternatively, thaw 1 ml recombinant human vitronectin (VTN‐N) at 4°C and divide into four 250 µl‐aliquots. Store in –80°C freezer.
2b Use 48 ml DMEM/F12 to dilute each 250‐µl aliquot and add 1 ml/well of mixture to a 6‐well plate.
3b Allow plates to rest for 2 hr at room temperature before use.
  Coated plates can be stored at 4°C for up to 1 month before use.
  Proceed to step 7.

### Thawing and plating of hPSCs

7 Obtain a cryovial containing ∼1 × 10^6^ hiPSCs (JCAZ001) or hESCs (H7: WA07) from liquid nitrogen storage. Place in a 37**°**C water bath to thaw cells quickly and completely (no frozen pellet should be observed within the cryovial).
8 Remove the cryovial from the water bath, spray with 70% ethanol, and place in a biological safety cabinet.
9 Transfer the cells from the cryogenic vial into a 15‐ml conical tube containing 5 ml E8 medium with 10 µM Y‐27632 (E8 + 10 µM Y‐27632 medium).
10 Centrifuge the 15‐ml conical tube for 3 min at 200 × *g*, room temperature, ensuring that the rotor is properly balanced.
11 Meanwhile, obtain a Matrigel‐ or vitronectin‐coated 6‐well plate (step 6a or 3b) and place it into the sterile biological safety cabinet.
12 Remove medium from two wells of the 6‐well plate and replace with 1 ml/well of E8 + 10 µM Y‐27632medium.
13 When centrifugation of the 15‐ml conical tube (from step 10) has finished, aspirate the supernatant carefully and resuspend the cell pellet in 2 ml of E8 + 10 µM Y‐27632 medium by gently pipetting the suspension up and down four times with a 1000‐µl pipet.
14 Transfer the 1 ml of cell suspension mixture into each of the two wells of the 6‐well plate containing 1 ml E8 + 10 µM Y‐27632 medium (from step 12).
  Depending on the cell count within each cryovial, one cryovial typically contains enough cells to plate 2‐4 wells; if you wish to plate cells in more than two wells, resuspend the cell pellet in enough medium to allow 1 ml per well to be added—e.g., 4 wells = 4 ml.
15 After 24 hr, remove the E8 + Y‐27632 medium and replace with 2 ml fresh E8 medium (without Y‐27632).
16 Continue to culture the cells, feeding them every day with 2 ml E8 medium, until they reach ∼70%‐80% confluency.

### Cell passaging and maintenance

17 When cells reach ∼70%‐80% confluency, aspirate medium from one well of a 6‐well plate and add 1 ml Cell Dissociation Buffer (CDB).
18 Incubate at room temperature for 5‐10 min.
19 Confirm that cells are detaching from the well using a microscope (cells should have a bright white border).
20 Carefully aspirate the CDB buffer, leaving the cells still attached to the bottom of the well. Using a P1000 pipet, dislodge cells by pipetting 1 ml of E8 + 10 µM Y‐27632 onto the well surface. Repeatedly pipet up and down to cover the entire well surface. Confirm that the cells have been dislodged from the bottom of the culture well using a microscope.
21 Count cells using a hemocytometer (or other automated cell counter system) and reseed hPSCs at 1.2 × 10^5^ cells/well in a 6‐well plate containing E8 + 10 µM Y‐27632 medium.
  hPSCs can be passaged without Y‐27632 if the cell line has been adapted to passaging in its absence. In addition, some cells may grow at a faster rate and initial seeding density may be adjusted.
22 Place in the incubator overnight (37°C).
23 Continue to culture cells, feeding them daily with E8 medium, until hPSCs reach ∼70% confluency (∼4 days). Ensure E8 medium is brought to room temperature for 20 min before feeding cells.

### Cryopreservation of hPSCs

24 When cells reach 70%‐80% confluency, aspirate the E8 medium, add 1 ml of CDB to each well, and incubate at room temperature for 5‐10 min.
25 Confirm cell dissociation using a microscope.
26 Using a P1000 pipet, dissociate cells from the culture plates and suspend hPSCs in 1 ml Bambanker medium. Be careful to avoid bubbles.
27 Pipet the cell suspension into a cryovial suitable for freezing and store in a –80°C freezer overnight.
28 Remove the cryovials from the –80°C freezer and place in liquid nitrogen for long‐term storage.
  Cryopreserving ∼1‐2 million hPSCs in each cryovial will allow them to be thawed and plated in 2‐4 wells of a 6‐well plate.

## Basic Protocol 2 hPSC‐CM DIFFERENTIATION

**Figure 1:**
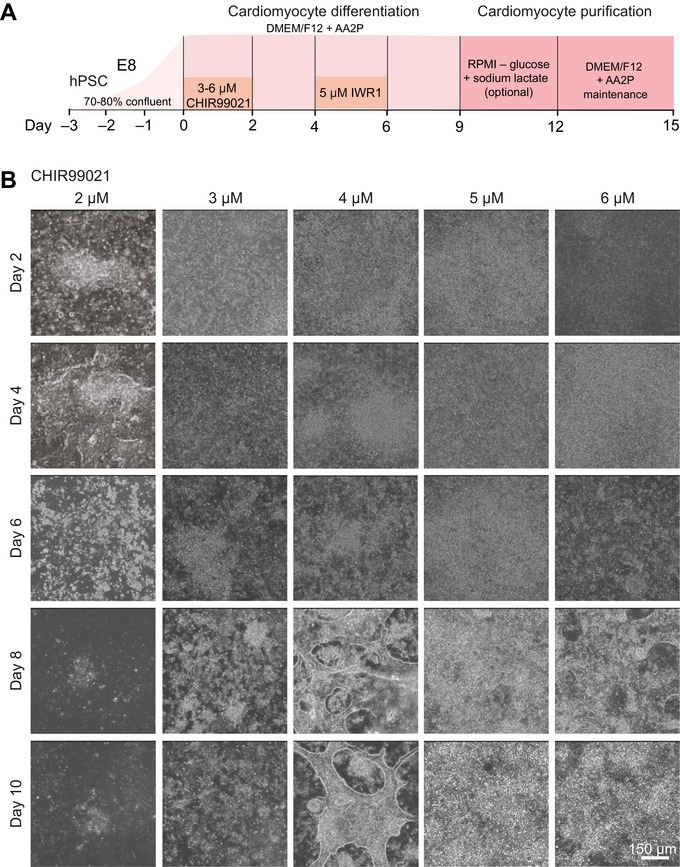
Schematic of hPSC‐directed differentiation and cellular morphological changes of hPSC‐CMs from hPSCs. (**A**) Schematic overview of the directed differentiation of hPSCs into hPSC‐CMs using the minimal‐component, protein‐free protocol outlined here. (**B**) Changes in cell morphology during various stages of the differentiation protocol. Days 0, 4, and 8 represent hPSCs, mesodermal/cardiac progenitors, and hPSC‐CMs, respectively. A concentration of 4 uM CHIR99021 at day 8‐10 optimally produced contracting cardiomyocytes cultures with limited non‐contracting cell types.

**Figure 2:**
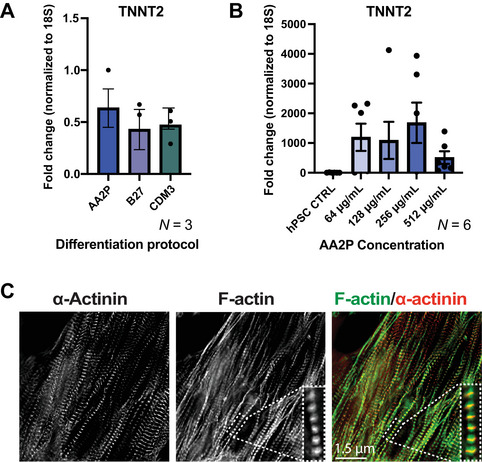
Three hPSC‐CM differentiation protocols performed in parallel demonstrate that AA2P alone successfully generates hPSC‐CMs at similar efficiencies. (**A**) Quantitative analysis via qPCR of TNNT2 fold change under varying differentiation conditions using either the l‐ascorbic acid 2‐phosphate (AA2P), B27 medium supplement, or chemically defined medium 3 (CDM3) protocol (*N* = 3). (**B**) Quantitative analysis via qPCR of cardiac troponin T (TNNT2) fold change with varying concentrations of AA2P (*N* = 6). (**C**) Representative immunofluorescence images of day 20 hPSC‐CMs stained with fluorescently conjugated phalloidin (F‐actin) and anti‐α‐actinin (a cardiac‐specific maker for the Z‐disc). Scale bar, 1.5 µM. Values are mean ± SD, *N* = 3‐6 independent cardiac differentiations, one‐way ANOVA with Tukey's multiple comparison test.

This section describes the process of differentiating hiPSCs and hESCs into hPSC‐CMs using a two‐component protocol involving l‐ascorbic acid 2‐phosphate (AA2P) dissolved in DMEM/F12 (as the basal medium) along with the biphasic Wnt modulation (sequential addition of CHIR99021 and IWR‐1 during various stages of differentiation) system (Fig. 1A). Of note, the concentration of CHIR99021 must be optimized for each cell line used, but the appropriate concentration is typically in the range of 3‐6 µM CHIR99021 (Fig. 1B). Many hPSC‐CM differentiation protocols utilize albumin (the CDM3 protocol [Burridge et al., 2014] or B27‐based protocol [Lian et al., 2012]) and/or complex mixtures containing various enzymes and fatty acids (e.g., B27 supplement); we have eliminated the use of albumin because it may contribute to variability in differentiation efficiency and it significantly increases the cost per differentiation. We have observed that even with many common differentiation components (and specifically albumin) removed, successful hPSC‐CM differentiation could still be achieved (Fig. 2A) at various AA2P concentrations (Fig. 2B). Therefore, by removing albumin we were able to achieve robust hPSC‐CM production that is protein‐ and xenobiotic‐free, as well as cost‐effective (Table 1).

**Table 1:** Minimal Differentiation Cost (Excluding Labor and Characterization) per 6‐well Plate (∼12 × 10^6^ hPSC‐CMs)

Components	Cost of components
AA2P medium	$0.20 × 10
CHIR99021	$0.15
IWR‐1	$0.11
Matrigel	$8.75/plate
6‐well plate	$1.10/plate
10‐ml serological pipets	$0.18 × 10
15‐ml conical tubes	$0.30 × 6
**Total cost**:	∼$15/6‐well plate of hPSC‐CMs

### Materials

l‐Ascorbic acid 2‐phosphate (AA2P) powder (Sigma‐Aldrich, cat. no. 49752‐100G)
Dulbecco's Modified of Eagle's Medium (DMEM/F12 50:50) with l‐glutamine and 15 mM HEPES (Corning, cat. no. 10‐092‐CM)
60% (w/w) sodium d‐lactate solution (Sigma Aldrich, cat. no. L4263‐500ML)
RPMI‐1640 without glucose (Life Technologies, cat. no. 11879020)
hPSCs from Basic Protocol 1
CHIR99021 (TOCRIS, cat. no. 4423)
IWR‐1‐endo (Selleckchem, cat. no. S7086)
TrypLE Express (Gibco, cat. no. 12605010)
Fetal bovine serum (Gibco, cat. no. 10437028)

1000‐, 200‐, 20‐, and 10‐µl pipets (Fisher Scientific, cat. no. 14‐285‐904)
Corresponding pipet tips (USA Scientific, cat. nos. 1111‐2020, 1110‐1000, 1111‐3000, and 1120‐1710)
1.5‐ml microcentrifuge tubes (Eppendorf, cat. no. 022363204)
–80°C freezer (Thermo Fisher Scientific, cat. no. TSX60086D)
1300 Series Class II, Type B2 Biological Safety Cabinets (Thermo Fisher Scientific, cat. no. 1310TS1)
Hemocytometer (Fisher Scientific, cat. no. 02‐671‐6)
Ibidi 35 mm µ‐dish (Ibidi Germany, 81156)

### Preparation of AA2P cardiomyocyte differentiation medium

1 Dissolve 6.4 g AA2P in 50 ml sterile water (128 mg/ml)
2 Once dissolved, sterile filter AA2P using a vacuum filter.
3 Generate 1‐ml aliquots, label, and store at –80°C.
4 Dissolve 1‐ml aliquot of AA2P into 1 liter DMEM/F12.

### Preparation of glucose starvation medium

5 Pipet 100 µl of 60% (w/w) sodium d‐lactate solution into 500 ml glucose‐free RPMI‐1640 medium.

### Differentiation of hPSCs into cardiomyocytes

6 Culture hPSCs in wells of a 6‐well plate for ∼3‐4 days to reach 70%‐80% confluency.
7 Day 0: When hPSCs reach 70%‐80% confluency, aspirate E8 medium from each well and replace with 2 ml/well of AA2P base medium supplemented with 4 µM CHIR99021.
  This concentration of CHIR99021 is optimized for the H7 hESC line. If using a different cell line, the concentration should be adjusted.
8 Day 1: Add 2 ml AA2P base medium to each well (do not remove the CHIR99021‐containing medium from the day before). Incubate for 24 hr at 37°C.
9 Day 2: Aspirate medium and replace with 2 ml fresh AA2P medium.
10 Day 3: Aspirate medium, replace with 2 ml fresh AA2P medium supplemented with 5 µm/well IWR‐1, and incubate plates for 24 hr at 37°C.
11 Day 4: The medium may turn yellow after 1 day in AA2P + IWR‐1. If this happens, add 2 ml AA2P base medium (do not aspirate the medium from the day before).
12 Days 5‐10: After cells have incubated for 48 hr in AA2P + IWR‐1, aspirate the medium and add 2 ml fresh AA2P medium to each well. Change the medium every other day until cardiomyocyte contraction is observed.
  Cardiomyocytes should begin contracting between 8 and 10 days.

### Glucose starvation

13 Days 10‐13: If hPSC‐CM differentiation is not pure (i.e., <80% of cells are contracting), glucose starvation may be performed. To do so, aspirate AA2P medium and replace with 4 ml of glucose starvation medium (from step 5). Keep cells in this medium for 3 days without replenishing.
  Although in our experience 3 days is sufficient in most cases, 4‐5 days may be required.
14 Days >13: Aspirate the glucose starvation medium and replace with 2 ml/well AA2P medium. Feed cells as required until the collection time point.

### Passaging of hPSC‐CMs

15 Aspirate AA2P medium from hPSC‐CMs.
16 Add 1 ml of TrypLE Express and incubate at 37°C for 10‐60 min. Confirm cell dissociation using a microscope.
17 Detach cells from the plate using a P1000 pipet and repeatedly pipet up and down 5‐10 times until cells are fully resuspended.
  If cells are difficult to detach, there will be significant cell death. Always confirm full dissociation before resuspending hPSC‐CMs.
18 Transfer the 1 ml of cell suspension into a 15‐ml conical tube containing 5 ml AA2P medium.
19 Centrifuge 5 min at 200 × *g*, room temperature.
20 Aspirate supernatant and resuspend the cell pellet in 5 ml AA2P medium.
21 Add 1 ml/well of cell suspension mixture to a Matrigel‐coated 6‐well plate (1‐2 × 10^6^ hPSC‐CMs) or an individual tissue culture dish (1 × 10^5^ for a 35‐mm ibidi well) with a coverslip insert (for immunocytochemistry; alternatively, a smaller amount can be added to the coverslip portion so that the cells settle just on that region). Recovery is typically 80% of the initial frozen concentration. hPSC‐CM contraction will be observed 3‐4 days after plating.
  Alternatively, with to increase cell viability during passage, 10% FBS can be added to the AA2P medium after TrypLE is removed during centrifugation and for the first 3 days in culture after plating. However, if cells will be used for downstream molecular or clinical applications, the addition of FBS is not recommended.

## Basic Protocol 3 CHARACTERIZATION OF hPSC‐CMs BY IMMUNOFLUORESCENCE (IF) IMAGING

After 8‐15 days, differentiated cardiomyocytes should be contractile and express cardiac‐specific proteins (e.g., cardiac α‐actinin and F‐actin, as shown in Fig. 2C). Immunofluorescence analysis may be performed, as described below, to confirm the presence of cell‐type‐specific features of differentiated cardiomyocytes.

### Materials

hPSC‐CM cultures from Basic Protocol 2
Phosphate‐buffered saline without calcium chloride and magnesium chloride (DPBS; Gibco, cat. no. 14190‐144)
4% formaldehyde (Fisher Scientific, cat. no. 427098)
Triton X‐100 Surfact‐Amps Detergent (Thermo Scientific, cat. no. 85111)
PBST: 0.1% Tween 20 in 1× phosphate‐buffered saline (PBS; e.g., Thermo Fisher Scientific, Gibco, cat. no. 10010002)
Blocking solution: 2% bovine serum albumin (BSA; Fisher BioReagents, cat. no. BP1600100)/22.5 mg/ml glycine (Sigma Aldrich, cat. no. G7403) in PBST (PBS + 0.1% Tween 20)
Primary antibody: Mouse monoclonal anti‐α‐actinin (1:200; EA‐53, Sigma‐Aldrich, St. Louis, MO)
Secondary antibody: Alexa Fluor 405‐conjugated goat anti‐mouse IgG (Thermo Fisher Scientific)
Texas Red‐X Phalloidin (1:100; Thermo Fisher Scientific cat. no. T7471)
VECTASHIELD Antifade Mounting Media (Vector Laboratories, cat. no. H‐1000‐10)

VWR Micro Cover Glasses, square, no. 1 (VWR Scientific, cat. no. 48366‐089)
Falcon Easy‐Grip Tissue Culture Dishes (Fisher Scientific cat. no. 08‐772A)
1000‐, 200‐, 20‐, and 10‐µl pipets (Fisher Scientific, cat. no. 14‐285‐904)
Pipet tips (USA Scientific, cat. nos. 1111‐2020, 1110‐1000, 1111‐3000, and 1120‐1710)
Fluorescence microscope (Nikon ECLIPSE Ts2)

1 Place coverslips coated with Matrigel into individual cell culture dish(es) as per the user's protocol.
2 Remove hPSC‐CMs from the incubator and aspirate AA2P medium.
3 Wash cells three times, 2 min each, in DPBS, each time by adding 1 ml/well DPBS (per well of a 35‐mm ibidi plate), letting it sit for 2 min at room temperature, and aspirating the liquid.
4 Aspirate DPBS and fix cells in 4% formaldehyde solution for 10 min at room temperature.
5 Remove 4% formaldehyde solution and wash three times, 5 min each, in DPBS.
6 Permeabilize cells with 0.2% Triton X‐100 in DPBS for 10 min at room temperature.
7 Aspirate Triton X‐100 and wash cells three times, 5 min each, in DPBS.
8 Incubate cells for 1 hr at room temperature in blocking solution.
9 Dilute primary antibodies in blocking solution according to the manufacturer's recommended dilution ratios.
10 Replace blocking solution with antibody solution and incubate overnight (4°C) in a humidified chamber.
11 In the morning, remove the antibody solution and wash three times, 5 min each, in PBST at room temperature.
12 Dilute secondary antibodies and Texas Red‐X Phalloidin in 0.2% BSA in PBST according to the manufacturer's recommended dilution ratios and incubate at room temperature for 1 hr in the dark.
13 Wash cells three times, 5 min each, in PBST at room temperature and then three times, 3 min each, in distilled, deionized water at room temperature.
14 Remove water and mount coverslip onto a microscope slide using antifade mounting medium.
15 Seal the coverslip using clear nail polish and store slide at 4°C overnight. Perform imaging the following day.

## COMMENTARY

### Critical Parameters

The successful generation of hPSC‐derived cardiomyocytes (hPSC‐CMs) relies on the preparation of precise concentrations of base medium and small‐molecule components. Secondary to this, careful observation of the color of the medium is required, as this can greatly influence the efficiency of hPSC‐CM differentiation. The color change of the medium is indicative of pH: AA2P base medium, when red, signifies physiological pH, and as CM differentiation continues, the medium color will change to orange/yellow, indicating that it is becoming more acidic. Although each iPSC line metabolizes medium at different rates, highly acidic conditions (e.g., bright yellow medium) may result in increased cell death. If significant cell death is observed after differentiation, the user may (1) add additional AA2P medium to each well during the second day of CHIR99021 or IWR1 treatment or (2) increase the total volume of base medium added to each well during each step of differentiation.

Lastly, attention should be paid to cells undergoing metabolic selection (with glucose‐free medium supplemented with sodium‐d‐lactate) to increase the purity of hPSC‐CMs. Many cell types require glucose (e.g., fibroblasts, iPSCs, and progenitor cell types) to survive. However, cardiomyocytes can utilize lactate as an alternative energy source (Tohyama et al., 2013). Variable levels of cell death will occur in non‐cardiomyocyte cells during glucose starvation, which typically lasts 3 days. However, additional days may be required to achieve the target purity goal (>80%), at which point observance of cell death in the background of cardiomyocytes should be performed daily if additional days of glucose starvation are required.

### Troubleshooting

For a list of problems, causes, and their possible solutions, see Table 2.

**Table 2:** Troubleshooting Guide for hPSC Cardiomyocyte Differentiation

Problem	Possible cause	Solution
Significant cell death following CHIR99021 addition	Concentration of CHIR99021 is too high or too low	Assess CHIR99021 concentrations in a range of 2‐12 µM
Low cardiomyocyte purity	Concentration of lactate selection medium is not optimal	100 µl sodium‐d‐lactate is used for purification; adjustments should be made to dilution and generation of stock medium as needed
	Days of exposure to lactate selection medium is not optimal	Increase the number of days hPSC‐CMs are exposed to lactate selection conditions.
	CHIR99021 concentration has not been optimized	Optimize CHIR99021 concentrations
iPSC detachment from the culture well	Rho kinase inhibitor not added for cell plating	Include Rho kinase inhibitor in medium for thawing and plating cells
	Matrigel plate made incorrectly	Ensure that Matrigel is kept ice cold throughout the plating procedure and is not older than the recommended time frame before use

### Understanding Results

This hPSC AA2P cardiomyocyte differentiation protocol (Fig. 1A) aims to overcome the challenges of current protocols that are time intensive and costly, do not generate hPSC‐CMs with sufficient purity, and, moreover, involve xenobiotic components that may affect the gene expression profiles of hPSCs, which may, in turn, confound downstream analysis. Therefore, careful selection of reagents and attention is required to produce hPSC‐CMs. Before beginning the process of differentiating hPSCs into cardiomyocytes, it is important to first optimize the concentration of CHIR99021 used to activate biphasic Wnt modulation, as this concentration may vary for each cell line used (Fig. 1B).

Many hPSC‐CM differentiation protocols rely on the use of recombinant human albumin and varying cocktails of chemicals to generate sufficient hPSC‐CMs. However, we have eliminated the use of these compounds because they may disrupt hPSC‐CM integrity and contribute to variability in differentiation efficiency. When hPSCs were differentiated into cardiomyocytes using CDM3 (which contains albumin, RPMI medium, and AA2P) versus AA2P alone, no statistical significance in the expression of the canonical cardiac marker, TNNT2, was observed (Fig. 2A). Furthermore, there was no significance in TNNT2 expression between the use of AA2P versus B27 supplement (which contains complex fatty acids, proteins, enzymes, etc.), indicating that AA2P alone is sufficient to generate hPSC‐CMs (Fig. 2A). To identify the optimal concentration of AA2P required for differentiation, a range of AA2P concentrations were tested (Fig. 2B). Although expression of TNNT2 could be achieved at 64, 128, 256, and 512 µg/ml, higher AA2P concentrations were not observed to enhance the expression levels of TNNT2. To validate differentiated cardiomyocytes, expressing cardiac‐specific proteins, we performed immunofluorescence microscopy on day 20 hPSC‐CMs in culture. Immunofluorescence images showed that the hPSC‐CMs expressed both filamentous actin (F‐actin) and cardiac α‐actinin (a marker of the Z‐disc) (Fig. 2C). Therefore, successful directed hPSC‐CM differentiation can be achieved using minimal components that are protein‐ and xenobiotic‐free. Moreover, the cost associated with hPSC cardiac differentiation is significantly reduced (Table 1), which can facilitate large‐scale projects.

### Time Considerations

The initial coating of tissue‐culture plates with either Matrigel or vitronectin, after thawing aliquots the previous night, should take the user ∼5‐30 min, depending on the number of plates being prepared. Once coated, the plates can rest for 16 hr at 4°C or 1 hr at room temperature before use. Next, thawing and plating hPSCs may take the user ∼10‐15 min, accounting for thawing of hPSCs, centrifugation, and plating cells onto coated tissue culture plates. Of note, this time may greatly vary depending on the number of cryovials and plates being prepared. Once plated, cells will rest for 24 hr and then require daily feeding with E8 medium. Daily feeding itself should take ∼5‐10 min; however, an additional 20 min should be considered for all steps to ensure the warming of medium solutions. After plating, cells may take several days to reach 70%‐80% confluency, at which point cell passage should be performed (∼15‐20 min per 6‐well plate) and later cryopreservation (∼10‐15 min per 6‐well plate).

Once AA2P differentiation medium and glucose starvation medium are prepared, hPSC‐CM differentiation will take ∼10 days for cardiomyocyte contraction to be observed. However, an additional 3‐5 days may be required to perform purification of hPSC‐CMs (as outlined in Fig. 1A). A total of 2 days (2‐3 hr/day) should be allocated for validation of hPSC‐CMs via immunofluorescence microscopy, with additional days anticipated to complete imaging and analysis.

### Author Contributions

**Jessika Iwanski**: Formal analysis; methodology; writing—original draft; writing—review and editing. **Odunayo Lawal**: Writing—original draft. **William Kwon**: Data curation. **Isabella Vazquez**: Data curation. **Jared Churko**: Funding acquisition; investigation; methodology; supervision; visualization; writing—review and editing.

### Conflict of Interest

The authors declare no conflict of interest.

## Data Availability

The data, tools, and materials are available from the corresponding author upon reasonable request.
